# Effect of implementation interventions on nurses’ behaviour in clinical practice: a systematic review, meta-analysis and meta-regression protocol

**DOI:** 10.1186/s13643-019-1227-x

**Published:** 2019-12-05

**Authors:** Guillaume Fontaine, Sylvie Cossette, Marc-André Maheu-Cadotte, Marie-France Deschênes, Geneviève Rouleau, Andréane Lavallée, Catherine Pépin, Ariane Ballard, Gabrielle Chicoine, Alexandra Lapierre, Patrick Lavoie, Jérémie Blondin, Tanya Mailhot

**Affiliations:** 1 Faculty of Nursing, Université de Montréal, Montréal, Canada; 2 Research Center, Montreal Heart Institute, Montréal, Canada; 3 Research Center, Université de Montréal Hospital Center, Montréal, Canada; 4 Center for Innovation in Nursing Education, Faculty of Nursing, Université de Montréal, Montréal, Canada; 5 Faculty of Nursing, Université Laval, Québec, Canada; 6 Research Center, CHU Sainte-Justine, Montréal, Canada; 7 Institute of Public Health Research, Université de Montréal, Montréal, Canada; 8 Research Center, Hôpital du Sacré-Coeur de Montréal, Montréal, Canada; 9 School of Librarianship and Information Science, Université de Montréal, Montréal, Canada; 10 Department of Pharmacy and Health Systems Sciences, Bouvé College of Health Sciences, Northeastern University, Boston, USA

**Keywords:** Behaviour change, Implementation strategy, Implementation intervention, Implementation science, Knowledge translation, Theory-based interventions, Nurses

## Abstract

**Background:**

Practitioner-level implementation interventions such as audit and feedback, communities of practice, and local opinion leaders have shown potential to change nurses’ behaviour in clinical practice and improve patients’ health. However, their effectiveness remains unclear. Moreover, we have a paucity of data regarding the use of theory in implementation studies with nurses, the causal processes—i.e. *mechanisms of action*—targeted by interventions to change nurses’ behaviour in clinical practice, and the constituent components—i.e. *behaviour change techniques*—included in interventions. Thus, our objectives are threefold: (1) to examine the effectiveness of practitioner-level implementation interventions in changing nurses’ behaviour in clinical practice; (2) to identify, in included studies, the type and degree of theory use, the mechanisms of action targeted by interventions and the behaviour change techniques constituting interventions and (3) to examine whether intervention effectiveness is associated with the use of theory or with specific mechanisms of action and behaviour change techniques.

**Methods:**

We will conduct a systematic review based on the Cochrane Effective Practice and Organization of Care (EPOC) Group guidelines. We will search six databases (CINAHL, EMBASE, ERIC, PsycINFO, PubMed and Web of Science) with no time limitation for experimental and quasi-experimental studies that evaluated practitioner-level implementation interventions aiming to change nurses’ behaviour in clinical practice. We will also hand-search reference lists of included studies. We will perform screening, full-text review, risk of bias assessment, and data extraction independently with the Covidence systematic review software. We will assess the quality of evidence using the GRADEpro software. We will code included studies independently for theory use (Theory Coding Scheme), mechanisms of action (coding guidelines from Michie) and behaviour change techniques (Behaviour Change Technique Taxonomy v1) with QSR International’s NVivo qualitative data analysis software. Meta-analyses will be performed using the Review Manager (RevMan) software. Meta-regression analyses will be performed with IBM SPSS Statistics software.

**Discussion:**

This review will inform knowledge users and researchers interested in designing, developing and evaluating implementation interventions to support nurses’ behaviour change in clinical practice. Results will provide key insights regarding which causal processes—i.e. mechanisms of action—should be targeted by these interventions, and which constituent components—i.e. behaviour change techniques—should be included in these interventions to increase their effectiveness.

**Systematic review registration:**

The protocol has been registered at the International Prospective Register of Systematic Reviews (PROSPERO; registration number: CRD42019130446).

## Background

Nurses represent the largest group of healthcare professionals that intervenes with patients in all sectors of health systems around the world [1]. Thus, nurses are often actively involved in initiatives aiming to improve service delivery to enhance patient outcomes [2]. However, changing nurses’ behaviour in clinical practice is a challenging and complex endeavor due to the influence of practitioner-level factors, including nurses’ motivational predispositions to change, and organizational-level factors [3, 4]. Multiple barriers specific to nursing practice, including lack of time, lack of organizational support, competing priorities and expanding workloads hinder the implementation of evidence-based nursing practices [5].

In the last decade, we have witnessed the emergence of implementation science, the scientific study of methods and theoretical approaches to improve health services and health through changes in healthcare professionals’ and organizations’ practices [6]. Implementation interventions have been associated with more effective health service delivery and improved health outcomes in several clinical practice settings [7–10]. A wide range of clinical behaviours have been targeted by these interventions, including medication prescribing, test ordering, disease screening and management, discharge planning and counseling [4, 9, 10]. Although nurses have frequently been the target of implementation interventions, we know little about the effectiveness, theoretical underpinnings and components of these interventions.

### Description of implementation interventions

An implementation intervention is defined as any strategy or program ‘aimed at increasing the use of research-based knowledge in healthcare practice (p. 2)’ [11]. Implementation interventions targeting specifically healthcare professionals—i.e. practitioner-level implementation interventions—are described in the Cochrane Effective Practice and Organization of Care (EPOC) Group Taxonomy of Health System Interventions [12]. Examples of practitioner-level implementation interventions, also named *implementation strategies*, include audit and feedback, educational materials, educational games, communities of practice, local opinion leaders, printed educational materials and reminders [12].

### How implementation interventions might work

**Fig. 1:**
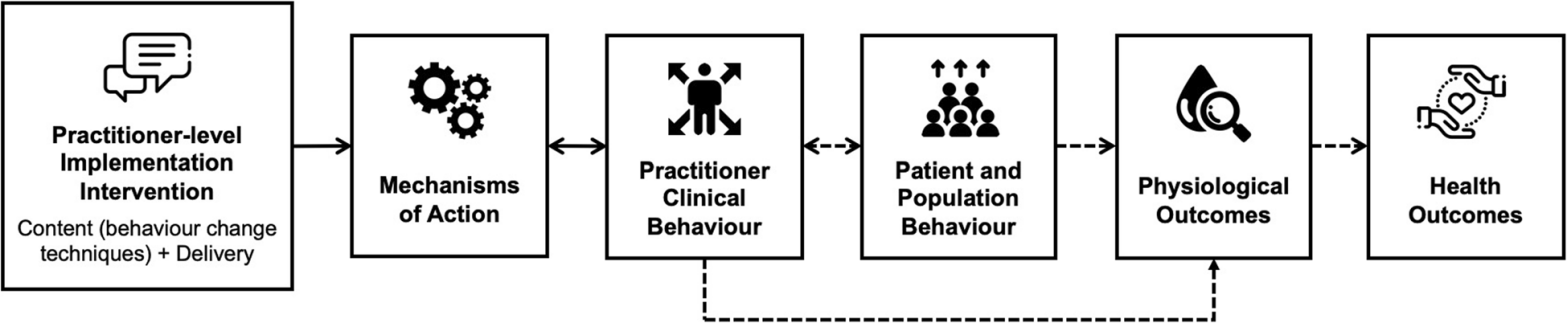
Causal modelling approach to the development of theory-based practitioner-level implementation interventions inspired by Hardeman [13], Michie [14] and Presseau [15]

Implementation interventions aim to ‘produce change in people’s behaviour or the environments in which they operate, or both (p. 2)’ [11]. Importantly, these interventions may aim for change at one or many levels (e.g. individual healthcare professionals, teams, organizations, system). Hereafter, we focus specifically on practitioner-level implementation interventions, which target behaviour change at the level of individual healthcare professionals and teams (i.e. nurses and teams of nurses in this review) (see Fig. 1).

Practitioner-level implementation interventions may be based on a wide range of theoretical approaches (i.e. theories, models, frameworks) [16]. Behavioural approaches to implementation science draw upon decades of research in social and health psychology [15]. Theories of behaviour and behaviour change (e.g. theory of planned behaviour, theory of interpersonal behaviour) appear particularly useful for predicting and explaining nurses’ behaviour in clinical practice. For instance, a researcher could investigate the extent to which nurses’ beliefs, attitudes and subjective norms concerning a clinical guideline predict/explain their adherence to this guideline in practice [16]. Thus, these theories may also be useful for selecting the potential *mechanisms of action* of behaviour change in nurses that will be targeted by an intervention to lead to successful implementation [17]. Mechanisms of action represent the causal processes through which an intervention, or a constituent component, affects nurses’ behaviour in clinical practice. These mechanisms of action ‘can be intrapersonal psychological processes of the individual (e.g. motivation, skills, attitudes) and/or characteristics of the social and physical environment (e.g. social support)’ [18]. Michie and colleagues have identified 26 mechanisms of action in theories of behaviour and behaviour change that may be targeted by interventions [18–20]. Describing the mechanisms of action targeted by implementation interventions could provide insight into the causal pathways leading to behaviour change in nurses.

‘Implementation intervention’ is an overarching term used to distinguish the intervention from its constituent components [15]. These components—the active ingredients of the intervention—can be described as *behaviour change techniques*. For instance, an implementation intervention based on audit and feedback may include multiple behaviour change techniques. Behaviour change techniques are “observable, replicable and irreducible components of an intervention designed to alter or redirect mechanisms of action that regulate behaviour; that is, a technique is proposed to be an ‘active ingredient’ (e.g. feedback, self-monitoring and reinforcement)” [21]. A taxonomy of 93 distinct behaviour change techniques, grouped into 16 clusters, has been developed by a Delphi consensus research method including a panel of international experts [21]. Some examples of the clusters of behaviour change techniques include ‘feedback and monitoring’, ‘comparison of outcomes’ and ‘repetition and substitution’. Describing behaviour change techniques included in implementation interventions would be useful for reporting, replicating and synthesizing evidence.

Thus, it is hypothesized that implementation interventions include multiple behaviour change techniques altering different mechanisms of action to effect behaviour change in nurses. For example, the implementation intervention ‘printed educational materials’ may include behaviour change techniques such as ‘instruction on how to perform the clinical practice’ to alter mechanisms of action such as ‘knowledge’, ‘attitudes’, ‘beliefs’ and ‘perceived control’, to effect behaviour change in nurses [22]. The implementation intervention ‘local opinion leaders’, i.e. individuals using their influence to promote and effect behaviour change in clinical practice through leadership, will include other behaviour change techniques, such as ‘credible source in favour of the implementation of the clinical practice’ and target mechanisms of action such as ‘social norms’ [23].

### Why it is important to do this review

So far, implementation interventions have had inconsistent results with regard to changing nurses’ behaviour in clinical practice [3, 4, 24]. This may be explained by several factors. First, studies and reviews examining the effect of implementation interventions have often not addressed key mechanisms of action hypothesized be specific to nursing practice and the clinical context [3, 4, 7–10]. Second, it appears that multiple interventions have been theory-inspired rather than theory-based. Indeed, researchers often rely on theoretical approaches only for some part of their intervention rather than adopt a systematic, theory-based intervention development process [17]. Thus, it appears important to examine the type and degree of theory use (e.g. reference to underpinning theory, measurement of constructs) in implementation interventions targeting nurses in addition to the effectiveness of such interventions [17, 25]. Third, there has been little research regarding the optimal constituent components—i.e. the behaviour change techniques—of implementation interventions targeting nurses. This limits our ability to make recommendations regarding intervention characteristics likely to lead to successful implementation in nurses.

To our knowledge, no review has looked into the effectiveness, theoretical underpinnings (i.e. theory use, mechanisms of action targeted) and behaviour change techniques of practitioner-level implementation interventions aiming to change nurses’ behaviour in clinical practice and, ultimately, improve patient outcomes. Thus, our objectives are threefold:

1. To examine the effectiveness of practitioner-level implementation interventions in changing nurses’ behaviour in clinical practice and in improving patient outcomes;
2. To identify:
  - The types—i.e. individual theory items, categories of theory use—and degree—i.e. total theory use score—of theory use in the development and evaluation of these interventions according to the Theory Coding Scheme [25];
  - The causal processes—i.e. mechanisms of action—targeted by these interventions to bring about behaviour change in nurses according to guidelines of Michie and colleagues [18–20];
  - The constituent components—i.e. behaviour change techniques—included in these interventions according to the Behaviour Change Technique Taxonomy v1 [21];
3. To examine whether using theory, targeting specific mechanisms of action and including specific behaviour change techniques increase implementation intervention effectiveness in changing nurses’ behaviour in clinical practice.

## Methods

This systematic review protocol is based on the Effective Practice and Organization of Care (EPOC) Cochrane Group guidelines [26, 27] and reported according to the Preferred Reporting Items for Systematic review and Meta-Analysis Protocols (PRISMA-P) Checklist [28] (see Additional file 1). This protocol was prospectively registered on the International Prospective Register of Systematic Reviews (PROSPERO; CRD42019130446; available from: https://www.crd.york.ac.uk/prospero/display_record.php?ID=CRD42019130446).

### Criteria for considering studies for this review

#### Types of studies

We will include all experimental studies (i.e. randomized controlled trials (RCTs), cluster RCTs, crossover RCTs) and quasi-experimental studies (i.e. non-randomized controlled trials, cluster non-randomized controlled trials). We will exclude all qualitative, cross-sectional, observational studies, case reports, discussion papers, editorials, knowledge syntheses, dissertations and theses. We will only include studies published in English or in French, regardless of the geographic location, in a peer-reviewed journal and in peer-reviewed conference proceedings.

#### Types of participants

We will include studies conducted with registered nurses (RNs), clinical nurse specialists (CNSs), nurse practitioners (NPs), licensed practical nurses (LPNs) or registered practical nurses (RPNs). We will include studies conducted in any type of clinical setting (e.g. hospitals, ambulatory clinics, community health centres). We will exclude studies including other groups of healthcare professionals and/or undergraduate nursing students.

#### Types of interventions

We will include studies reporting practitioner-level implementation interventions targeting nurses. We define a ‘practitioner-level implementation intervention’ as any strategy aimed at increasing the use of research-based knowledge in healthcare through changes in nurses’ clinical practice [6, 29]. More specifically, we will consider for inclusion studies which report an intervention including at least one implementation strategy targeting specifically nurses as described in a subsection of the Cochrane Effective Practice and Organization of Care (EPOC) Group Taxonomy of Health System Interventions [12] (see Additional file 2). We will include studies combining multiple implementation strategies listed in the EPOC Group Taxonomy of Health System Interventions. However, we will exclude studies including financial interventions, patient-oriented organizational interventions, structural organizational interventions and regulatory interventions, which are beyond the scope of this review.

We will include studies with all types of comparator(s).

### Types of outcome measures

#### Primary outcome

We will include studies reporting on at least one outcome related to a change in nurses’ behaviour in clinical practice. More specifically, we will include studies reporting an objective measure of nurses’ behaviour (e.g. clinical interventions reported in patients’ medical files, number of tests ordered) or a subjective measure of nurses’ behaviour (e.g. self-reported performance of clinical interventions).

#### Secondary outcomes

We will also collect data related to the following outcomes:

- Other outcomes in nurses
  - Objective or subjective measures of nurses’ *intention* to change behaviour in clinical practice and other hypothesized mechanisms of action, including knowledge, attitudes, beliefs, subjective norms and skills.
- Patient health behaviour, health status and well-being
  - Objective measures of patient health behaviour, health status and well-being, including physical health and treatment outcomes, psychological health and psychosocial outcomes, as long as they can be associated with nurses’ interventions performed in clinical practice.

### Search methods for identification of studies

#### Electronic searches

We developed the search strategy with a graduate student in librarianship and information science (JB). The search strategy was then validated by an experienced librarian. It includes a combination of three major concepts: (1) implementation interventions; (2) nurses; (3) study design (see Additional file 3). We first developed the search strategy for PubMed (see Additional file 4), then tailored it to each database. We refined the search strategy over a period of 2 months to ensure specificity, sensibility and replicability in all databases. The search strategy targets six databases:

- Cumulative Index to Nursing and Allied Health Literature (CINAHL), via EBSCOhost (1980 to present);
- Excerpta Medical Database (EMBASE), via Ovid SP (1947 to present);
- Education Resources Information Center (ERIC), via Ovid SP (1966 to present);
- PsycINFO, via APA PsycNet (1967 to present);
- PubMed (including MEDLINE), via NCBI (1946 to present);
- Web of Science—Science Citation Index (SCI) Expanded and Social Sciences Citation Index (SSCI), via Clarivate Analytics (1900 to present).

#### Searching other resources

Using a snowball method, we will manually screen the reference list of included studies to identify additional studies by looking at titles. In addition, we will search the Cochrane Database of Systematic Reviews (CDSR) and Google Scholar for related systematic reviews to find additional studies.

## Data collection and analysis

The different stages of data collection will be conducted by review authors in teams of two. Five teams of two were formed: team A (GF and CC), team B (AB and ALavallée), team C (MAMC and CP), team D (GR and GC) and team E (ALapierre and MFD) (see Table 1). The teams were formed based on the experience of each review author in a particular field (e.g. screening titles and abstracts, assessing risk of bias, coding studies using qualitative research software).

**Table 1:** Review stages and review teams involved

Review stages	Review teams
Selection of studies	Teams A, B, C, D and E
Data extraction	Teams A, B, C, D and E
Risk of bias assessment	Teams A, B, C, D and E
Theory coding	Teams A, D and E
Mechanisms of action coding	Teams A, B, C, D and E
Behaviour change technique coding	Teams A, B, C, D and E

### Selection of studies

We will manage the records obtained with the search strategy with the Covidence systematic review software v1430 (Veritas Health Innovation, Melbourne, Australia; www.covidence.org) [30]. Covidence is the primary screening and data extraction tool for Cochrane authors, streamlining the production of intervention reviews. Ten review authors, in teams of two, will independently screen all titles and abstracts retrieved by the search strategy and apply the eligibility criteria. We will conduct a full-text review for the citations who will be rated as relevant, potentially relevant or with unclear relevance by at least one of the two reviews authors. Ten review authors, in teams of two, will independently screen full-text articles and identify studies for inclusion and identify and record reasons for the exclusion of the ineligible studies. At any time during the review process, we will resolve disagreements through discussion and consensus. An author not involved in the study selection process will make a decision in case of a persistent disagreement. We will record the process of study selection in a PRISMA flow chart [31].

### Data extraction and management

A modified version of the Cochrane EPOC Review Group data collection form [32] was developed specifically for this review. This form will be iteratively validated by the whole team to ensure its completeness and clarity. Before data collection, we will calibrate our data collection form on a random sample of five full-text articles. The data collection form will be revised for clarity, as needed. Subsequently, ten review authors, in teams of two, will conduct all data collection for each study independently. We will collect data at the following levels:

- Study level: study design, year of study conduct, sample size, power analysis (yes/no), type of randomization, setting, country of study conduct, study funding source(s) and contact author;
- Participant level: type and number of participants, inclusion criteria, withdrawals and exclusions (loss to follow-up), age, sex, level of instruction, practice setting;
- Intervention level: implementation strategies included in each intervention according to the EPOC Taxonomy (see Additional file 2), framework(s), model(s) or theory(ies) underlining the intervention, clinical topic(s), target clinical practice(s) in nurses, timing (frequency, duration of the intervention), mode of delivery, providers, economic variables (e.g. intervention cost), description of control group(s) intervention(s);
  - The types—i.e. individual theory items, categories of theory use—and degree—i.e. total theory use score—of theory use, the mechanisms of action targeted, and the behaviour change techniques included in implementation interventions will be identified during a coding phase after data extraction;
- Outcome level: name, time points measured, definition, unit of measurement, scales, validation of measurement tool, missing data, results according to our primary and secondary outcomes, intention to treat (yes/no).

### Theory coding

We will conduct a theoretical analysis of included studies using an amended version of the Theory Coding Scheme [25]. As Garnett et al. [33] suggested, we removed the items ‘quality of measures’ and ‘randomization of participants to condition’ because they relate to methodological issues rather than theory use. The amended Theory Coding Scheme has a total of 17 items (three of which have sub-items) (see Additional file 5). Six review authors in teams of two will code each study independently using QSR International’s NVivo version 12 qualitative data analysis software [34] for specifying if each Theory Coding Scheme item is present (1) or absent (0). We will resolve differences through discussion, and we will involve another review author if a consensus is not reached. Rounds of testing will be performed initially until the inter-rater reliability (IRR) reaches a substantial level of agreement (prevalence-adjusted bias-adjusted kappa (PABAK) statistic greater or equal to .70 [35, 36]). A total theory use score will be calculated (i.e. the sum of all 17 items and sub-items, which will result in a maximum possible score of 22). A higher score will be indicative of a highest degree of theory use.

### Mechanism of action coding

We will code the mechanisms of action of behaviour change in clinical practice targeted by implementation interventions using coding guidelines from Michie and colleagues [18–20]. We will use the labels and definitions of the 26 mechanisms of action listed on the Theory and Technique Tool (www.theoryandtechniquetool.humanbehaviourchange.org/tool) associated with the three publications mentioned above [18–20] (see Additional file 6). Each mechanism of action will be coded as either present (1) or absent (0) in the experimental and comparator interventions. To be coded as ‘present’, the mechanism of action will have to be explicitly mentioned/used to select or develop intervention techniques (as specified in the item 5 of the Theory Coding Scheme [25]). Mechanism of action coding will be conducted using QSR International’s NVivo version 12 qualitative data analysis software [34]. Ten review authors in teams of two will code each study for mechanisms of action independently, differences will be resolved through discussion and we will involve another review author if a consensus is not reached. Rounds of testing will be performed initially until the IRR reaches a substantial level of agreement (PABAK greater or equal to .70 [35]).

### Behaviour change technique coding

We will use the labels, definitions and examples of the 93 behaviour change techniques included in the Behaviour Change Technique Taxonomy v1 [21] to code studies for behaviour change techniques. In addition, we will use the coding tool developed by Pearson, Byrne-Davis [37] illustrating behaviour change techniques applied to health professional training. A coding manual and instructions will be given to review authors. Review authors involved in the behaviour change technique coding will complete the Behaviour Change Technique Taxonomy Online Training (www.bct-taxonomy.com) prior to coding. The training, lasting approximately 6 h, is a resource where researchers can familiarize themselves with behaviour change technique labels, definitions and examples, and learn how to accurately, reliably and confidently apply the taxonomy. When review authors identify a behaviour change technique in the experimental intervention or in the comparator intervention, they will code the behaviour change technique as either present in all probability (+) or present beyond all reasonable doubt (++). Behaviour change technique coding will be conducted using NVivo version 12 [34]. Ten review authors in teams of two will code each study for behaviour change techniques independently, differences will be resolved through discussion and we will involve another review author if a consensus is not reached. Rounds of testing will be performed initially until the IRR reaches a substantial level of agreement (PABAK greater or equal to .70 [35]).

### Assessment of risk of bias in included studies

Ten review authors in teams of two will assess risk of bias independently for each study using the criteria outlined in the revised Cochrane Collaboration Risk of Bias Tool (RoB 2.0) [38]. Any disagreement will be resolved by discussion or by involving another review author. For individually randomized trials (including crossover trials) and non-randomized controlled trials, we will assess the risk of bias according to the following domains: (1) bias arising from the randomization process; (2) bias due to deviations from intended interventions; (3) bias due to missing outcome data; (4) bias in measurement of the outcome; (5) bias in selection of the reported result. For cluster-randomized trials, we will include an additional domain: (1b) bias arising from identification or recruitment of individual participants within clusters. Non-randomized studies will be considered at high risk of bias. We will summarize the ‘risk of bias’ judgments across different studies for each of the domains listed using the risk of bias graph and the risk of bias summary. We will not exclude studies on the grounds of their risk of bias but we will report them when presenting the results of the studies.

### Unit-of-analysis issues

We anticipate the inclusion of cluster RCTs. Thus, we will evaluate the analysis methods of these studies by determining the level of analysis and if statistical corrections were used (e.g. generalized estimating equations). We will conduct analyses adjusting for clustering if we observe unit-of-analysis issues by dividing the original sample size by the design effect, as suggested by the *Cochrane Handbook for Systematic Reviews of Interventions* [27]. For studies with multiple intervention groups, we will include each pairwise comparison relevant to this review separately, but with shared intervention groups divided out approximately evenly among the comparisons [27].

### Dealing with missing data

We will contact investigators to obtain missing data when necessary. In the case where investigators do not answer our request, data imputation will be performed using the statistical formulas recommended by the *Cochrane Handbook for Systematic Reviews of Intervention* [27] when applicable. In the case where missing outcome data cannot be obtained and data imputation cannot be performed, we will exclude the study for the outcome in question.

### Assessment of heterogeneity

We will assess heterogeneity by examining the characteristics of included studies, the similarities and disparities between the types of participants, the types of interventions and the types of outcomes. We will then use the chi-square statistic and the *I*^2^ to assess statistical heterogeneity for analyses including two studies or more within the Review Manager (RevMan) software (version 5.3. Copenhagen: The Nordic Cochrane Centre, The Cochrane Collaboration, 2014). For the chi-square statistic, we will use a statistical significance level (*p* value) of 0.10 instead of the conventional level of 0.05, as this test is known to have low statistical power [27]. A statistically significant result will indicate a problem of heterogeneity [27]. For the *I*^2^ statistic, as suggested by Higgins et al. [27], we will interpret the values as follows: 0–40%, might not be important; 30–60%, may represent moderate heterogeneity; 50–90%, may represent substantial heterogeneity and 75–100%, considerable heterogeneity.

### Assessment of reporting biases

We will assess reporting biases using funnel plots if more than 10 studies are included in the meta-analysis for a specific outcome. We will follow the guidelines regarding funnel plot asymmetry as described in the *Cochrane Handbook for Systematic Reviews of Interventions* [27]. We will also perform Egger’s regression to further assess a publication bias [27, 39]. Egger’s regression is a linear type of regression between each study standard normal deviate (i.e. mean difference between the groups in a single pairwise comparison divided by its standard error) and its precision (i.e. inverse of the standard error). Egger’s regression will be performed using IBM SPSS Statistics (Version 25, IBM Corporations). An asymmetrical funnel plot at visual inspection and a *p* value ≤ to 0.05 for the constant of the regression will be considered as indicative of publication bias.

### Data synthesis

#### Descriptive synthesis

We will synthesize the characteristics of included studies at four levels—i.e. study level, participant level, intervention level, outcome level—in table format. We will quantify the types—i.e. individual theory items, categories of theory use—and degree—i.e. total theory use score—of theory use, the types, categories and number of identified mechanisms of action, and the type and number of identified behaviour change techniques across studies.

#### Quantitative synthesis

All summary intervention effects estimates will be presented using a random-effects model using a 95% confidence interval (CI) as we anticipate clinical and methodological heterogeneity across included studies. For continuous outcomes, we will analyze data using the standardized mean difference (SMD) since it is not expected studies will have the same outcome measures/scales to evaluate implementation. We will ensure that an increase in scores for continuous outcomes can be interpreted in the same way for each outcome, and report where the directions will be reversed if this is necessary. For dichotomous outcomes, we will pool events between groups across studies using risk ratios and 95% CIs.

We will undertake meta-analyses that will compare changes between intervention and control participants in primary and secondary outcomes only if: (1) the implementation interventions, targeted clinical practices and the underlying clinical question are similar enough for pooling to make sense; (2) there is at least two studies available for each outcome of interest. Meta-analyses will be conducted in RevMan version 5.3 software (Copenhagen: The Nordic Cochrane Centre, The Cochrane Collaboration, 2014) [40]. The significance of the effect sizes will be determined using Cohen’s classification (< 0.2 = negligible; 0.2—0.49 = small; 0.5—0.8 = moderate; > 0.8 = large) [41]. We will define a statistically significant result by a two-sided alpha of 0.05. If it is not possible to conduct a meta-analysis, we will present a narrative summary of the results.

#### Meta-regression

We will undertake random-effects meta-regression analyses if at least 10 studies report enough data to compute a SMD regarding the primary outcome (clinical practice change). We will conduct meta-regression analyses to: (1) examine the association between the Theory Coding Scheme covariates (i.e. individual theory items, categories of theory use and total theory use) with intervention effectiveness; (2) examine the association between type, categories and number of mechanisms of action with intervention effectiveness; (3) examine the association between type and number of behaviour change techniques with intervention effectiveness.

Meta-regression analyses will serve to investigate unexplained heterogeneity in the SMDs between studies. Each study will be weighted in the regression models using the inverse of its variance; studies with the lowest amount of variance will be given a bigger weight in the regression model than those with the largest amount of variance. The association between each variable of interest and the primary outcome will be illustrated in table format where, for each variable, we will report its regression coefficient (B), standard error, 95% CI and statistical significance. Meta-regression analyses will be conducted in IBM SPSS Statistics version 25.0 [42]. Wilson’s SPSS macros will be used to build all regression models [43, 44].

### ‘Summary of findings’ table and GRADE

We will create a ‘summary of findings’ table for the main intervention comparison(s) and include the most important outcomes (e.g. nurses’ behaviour in clinical practice) to draw conclusions about the certainty of the evidence. Two review authors will assess the quality of the evidence independently for each outcome according to the five domains (risk of bias, inconsistency, indirectness, imprecision, publication bias) established by the Grading of Recommendations Assessment, Development, and Evaluation (GRADE) guidelines [45]. Review authors will use the GRADE profiler Guideline Development Tool software (GRADEpro; 2015, McMaster University and Evidence Prime Inc.) [46], based upon the data extracted with the data collection checklist.

### Subgroup analysis and investigation of heterogeneity

We plan to carry out subgroup analyses to investigate heterogeneity when ten or more studies are available in the underlying outcome. If there are a sufficient number of studies, we will explore the following potential effect modifiers:

- Implementation intervention types according to EPOC taxonomy [12];
- Practice setting;
- Clinical practice(s) targeted in nurses;
- Study design.

### Sensitivity analysis

We will conduct a sensitivity analysis by excluding studies deemed at high risk of bias. We will also conduct a sensitivity analysis to exclude studies with imputed data.

## Discussion and dissemination

Results of this systematic review, meta-analysis and meta-regression will inform knowledge users (e.g. practitioners, policy-makers) and researchers regarding the effectiveness of practitioner-level implementation interventions in changing nurses' behaviour in clinical practice. In addition, data regarding the theory use, targeted mechanisms of action and included behaviour change techniques in studies will be useful for reporting, replicating and synthesizing evidence. Results will be disseminated through publications, conference presentations, website postings and interactive knowledge exchange events with key stakeholders.

This review has potential limitations. First, this review will build exclusively on published studies, whereas unpublished studies, grey literature and non-peer-reviewed literature will be excluded. Although including unpublished, grey and non-peer-reviewed literature has potential benefits in terms of comprehensiveness, it can introduce bias in the results of the systematic review and meta-analysis. Unpublished studies are usually of lower methodological quality than published studies [47]. Second, we anticipate that outcome measures for nurses’ behaviour in clinical practice will vary significantly across studies. Thus, we will conduct a meta-analysis using the SMD. This will allow us to standardize the results of studies to a uniform scale before pooling them. However, this method also has downsides since it assumes that the differences in standard deviations among studies reflect differences in measurement scales and not differences in variability among study populations [27]. Review authors deemed the use of the SMD appropriate for this review since it focuses on nurses, minimizing the risk of bias. Third, this review focuses exclusively on practitioner-level implementation interventions and their effect on nurses’ behaviour in clinical practice and patient outcomes. Other types of implementation interventions (e.g. financial interventions, patient-oriented organizational interventions, structural organizational interventions, regulatory interventions) may have important effects on nurses’ behaviour in clinical practice. However, we believe these interventions differ in scope and deserve their own review.

## Supplementary information

**Additional file 1.** PRISMA-P ChecklistR1.

**Additional file 2.** EPOC TaxonomyR1.

**Additional file 3.** Concept PlanR1.

**Additional file 4.** PubMed Search StrategyR1.

**Additional file 5.** Theory Coding SchemeR1.

**Additional file 6.** List of Mechanisms of ActionR1.

## Data Availability

No additional data are available.

## Abbreviations

CI: Confidence interval
CINAHL: Cumulative Index to Nursing & Allied Health Literature
CNSs: Clinical nurse specialists
EMBASE: Excerpta Medical Database
EPOC: Effective Practice and Organization of Care
ERIC: Education Resources Information Center
GRADE: Grading of Recommendations Assessment, Development, and Evaluation
IRR: Inter-rater reliability
LPNs: Licensed practical nurses
NCBI: National Center for Biotechnology Information
NPs: Nurse practitioners
PABAK: Prevalence-adjusted bias-adjusted kappa
PRISMA-P: Preferred reporting items for systematic review and meta-analysis protocols
RCT: Randomized controlled trial
RNs: Registered nurses
RoB: Risk of bias
RPNs: Registered Practical Nurses
SCI: Science Citation Index
SPSS: Statistical Package for the Social Sciences
SSCI: Social Sciences Citation Index
