# Supplementary material for: Effect of implementation interventions on nurses’ behaviour in clinical practice: a systematic review, meta-analysis and meta-regression protocol
Source: Syst Rev. 2019 Dec 5;8:305. doi: 10.1186/s13643-019-1227-x (PMC6896305; doi:10.1186/s13643-019-1227-x)
Supplement: Supplementary file 2 — Additional file 2. EPOC TaxonomyR1. [file 13643_2019_1227_MOESM2_ESM.docx]

**ADDITIONAL FILE 2**

**EPOC Taxonomy of Implementation Strategies Targeting Healthcare Professionals**

- Source: Effective Practice and Organization of Care (EPOC). *EPOC Taxonomy; 2015*. Available at: <https://epoc.cochrane.org/epoc-taxonomy>

| **#** | **IMPLEMENTATION STRATEGIES** | **DEFINITION** |
| --- | --- | --- |
| **1** | **Audit and Feedback** | A summary of health workers’ performance over a specified period of time, given to them in a written, electronic or verbal format. The summary may include recommendations for clinical action. |
| **2** | **Clinical Incident Reporting** | System for reporting critical incidents. |
| **3** | **Monitoring the Performance of the Delivery of Healthcare** | Monitoring of health services by individuals or healthcare organizations, for example by comparing with an external standard. |
| **4** | **Communities of Practice** | Groups of people with a common interest who deepen their knowledge and expertise in this area by interacting on an ongoing basis. |
| **5** | **Continuous Quality Improvement** | An iterative process to review and improve care that includes involvement of healthcare teams, analysis of a process or system, a structured process improvement method or problem solving approach, and use of data analysis to assess changes. |
| **6** | **Educational Games** | The use of games as an educational strategy to improve standards of care. |
| **7** | **Educational Materials** | Distribution to individuals, or groups, of educational materials to support clinical care, i.e., any intervention in which knowledge is distributed. For example, this may be facilitated by the internet, learning critical appraisal skills; skills for electronic retrieval of information, diagnostic formulation; question formulation. |
| **8** | **Educational Meetings** | Courses, workshops, conferences or other educational meetings. |
| **9** | **Educational Outreach Visits, or Academic Detailing** | Personal visits by a trained person to health workers in their own settings, to provide information with the aim of changing practice. |
| **10** | **Clinical Practice Guidelines** | Clinical guidelines are systematically developed statements to assist healthcare providers and patients to decide on appropriate health care for specific clinical circumstances' (US IOM). |
| **11** | **Inter-Professional Education** | Continuing education for health professionals that involves more than one profession in joint, interactive learning. |
| **12** | **Local Consensus Processes** | Formal or informal local consensus processes, for example agreeing a clinical protocol to manage a patient group, adapting a guideline for a local health system or promoting the implementation of guidelines. |
| **13** | **Local Opinion Leaders** | The identification and use of identifiable local opinion leaders to promote good clinical practice. |
| **14** | **Managerial Supervision** | Routine supervision visits by health staff. |
| **15** | **Patient-Mediated Interventions** | Any intervention aimed at changing the performance of healthcare professionals through interactions with patients, or information provided by or to patients. |
| **16** | **Public Release of Performance Data** | Informing the public about healthcare providers by the release of performance data in written or electronic form. |
| **17** | **Reminders** | Manual or computerized interventions that prompt health workers to perform an action during a consultation with a patient, for example computer decision support systems. |
| **18** | **Routine Patient-Reported Outcome Measures** | Routine administration and reporting of patient-reported outcome measures to providers and/or patients. |
| **19** | **Tailored Interventions** | Interventions to change practice that are selected based on an assessment of barriers to change, for example through interviews or surveys. |
