# Supplementary material for: Effect of implementation interventions on nurses’ behaviour in clinical practice: a systematic review, meta-analysis and meta-regression protocol
Source: Syst Rev. 2019 Dec 5;8:305. doi: 10.1186/s13643-019-1227-x (PMC6896305; doi:10.1186/s13643-019-1227-x)
Supplement: Supplementary file 3 — Additional file 3. Concept PlanR1. [file 13643_2019_1227_MOESM3_ESM.docx]

**ADDITIONAL FILE 3**

**Concept Plan**

| **Implementation Intervention** | | **Nurses** | **Study Design** |
| --- | --- | --- | --- |
| - Diffusion of innovation - Integrated knowledge - Integrated knowledge translation - Knowledge broker - Knowledge mobilization - Knowledge transfer - Knowledge translation - Knowledge uptake - Medical translation - Translational research - Knowledge dissemination - Knowledge exchange - Reminders - Implement - Implementation - Implementing - Local opinion leader | - Local consensus processes - Community of practice - Clinical practice guidelines - Managerial supervision - Audit - Feedback - Training - Learning - Clinical incident reporting - Serious game - Educational - Inter-professional education - Academic detailing - E-learning - Web-based learning - Computer-based learning | - Nurse - Nurse employee - Nurse personnel - Nurse staff - Nursing personnel - Nursing staff | - Evaluation Studies - Clinical Trial - Clinical study - Guideline |

| **Limit terms (NOT)** | | **Other limits** |
| --- | --- | --- |
| Terms | Study Design | Language |
| - Student - Undergraduate - Cross-sectional studies (MH) - Qualitative research (MH) - Observational studies (MH) | - Qualitative research - Editorial - Observational study - Case report - Review | - English - French |
