# Supplementary material for: Effect of implementation interventions on nurses’ behaviour in clinical practice: a systematic review, meta-analysis and meta-regression protocol
Source: Syst Rev. 2019 Dec 5;8:305. doi: 10.1186/s13643-019-1227-x (PMC6896305; doi:10.1186/s13643-019-1227-x)
Supplement: Supplementary file 4 — Additional file 4. PubMed Search StrategyR1. [file 13643_2019_1227_MOESM4_ESM.docx]

**ADDITIONAL FILE 4**

**PubMed search strategy**

Date of preliminary search (via NCBI): April 15 2019

| **Search** | **Query** | Items found |
| --- | --- | --- |
| #1 | (Translational Medical Research[MH] OR Knowledge Management[MH] OR Knowledge[MH] OR Implementation Science[MH] OR Information Dissemination[MH] OR Clinical audit[MH] OR Feedback[MH]  OR Reminder Systems[MH] OR Health plan Implementation[MH] OR Practice Guideline[MH] OR education[MH] OR learning[MH]) OR ("Knowledge transfer"[TIAB] OR "Knowledge transfers"[TIAB] OR "knowledge translations"[TIAB] OR "Knowledge Translation"[TIAB] OR "Knowledge mobilisation"[TIAB] OR "Knowledge mobilisations"[TIAB] OR "Knowledge mobilization"[TIAB] OR "Knowledge mobilizations"[TIAB]  OR "knowledge uptake"[TIAB] OR "knowledge uptakes"[TIAB] OR "Knowledge management"[TIAB] OR "Knowledge dissemination"[TIAB] OR "Knowledge exchange "[TIAB] OR "Translation Research"[TIAB] OR "Translation Researches" OR "Translational Research"[TIAB] OR "Translational Researches"[TIAB] OR  Audit[TIAB] OR Audits[TIAB] OR Feedback*[TIAB] OR Reminder*[TIAB] OR Implement*[TIAB] OR "local opinion leader"[TIAB] OR "local opinion leaders"[TIAB] OR "local consensus process"[TIAB] OR "local consensus processes"[TIAB] OR "communities of practice"[TIAB] OR "community of practices"[TIAB] OR "community of practice"[TIAB] OR "managerial supervision"[TIAB] OR "clinical incident reporting"[TIAB] OR "academic detailing"[TIAB] OR "e-learning"[TIAB] OR "elearning"[TIAB] OR "web-based learning"[TIAB] OR "computer-based learning"[TIAB] OR "serious game"[TIAB] OR "serious games"[TIAB] OR education*[TIAB] OR course[TIAB] OR courses[TIAB] OR train*[TIAB] OR learn*[TIAB]) | 2 920 709 |
| #2 | (Nurses[MH] OR Nurs*[TIAB]) | 467 497 |
| #3 | ((eng[LA] OR fre[LA]) AND (clinical study[PT]  OR Guideline[PT] OR Evaluation Studies[PT])) | 1 078 255 |
| #4 | #1 AND #2 AND #3 | 11 679 |
| #5 | Students[MH] OR Education, Medical, Undergraduate[MH] OR Qualitative Research [MH] OR Cross-Sectional Studies[MH] OR observational study[PT] OR Systematic Reviews as Topic[MH] OR Observational Studies as Topic[MH] OR Systematic Review[PT] OR Student*[TIAB] OR School*[TIAB] OR undergrad*[TIAB] OR qualitativ*[TIAB] OR Editorial*[TIAB] OR Cross-sectional[TIAB] OR "Cross sectional"[TIAB] OR observational[TIAB] OR "systematic review"[TIAB] OR "systematic reviews"[TIAB] | 1 428 887 |
| #6 | #4 NOT #5 | 7 777 |
