# Supplementary material for: Effect of implementation interventions on nurses’ behaviour in clinical practice: a systematic review, meta-analysis and meta-regression protocol
Source: Syst Rev. 2019 Dec 5;8:305. doi: 10.1186/s13643-019-1227-x (PMC6896305; doi:10.1186/s13643-019-1227-x)
Supplement: Supplementary file 5 — Additional file 5. Theory Coding SchemeR1. [file 13643_2019_1227_MOESM5_ESM.docx]

**ADDITIONAL FILE 5**

**Theory Coding Scheme**

- Source: Michie, S., & Prestwich, A. (2010). Are interventions theory-based? Development of a theory coding scheme. *Health Psychology, 29*(1), 1-8. doi: [10.1037/a0016939](http://dx.doi.org/10.1037/a0016939)

| **#** | **Theory coding scheme item** | | **Item description** | |  |
| --- | --- | --- | --- | --- | --- |
| **1** | **Theory or model of behavior mentioned** | | Models or theories that specify relations among variables to explain or predict behavior are mentioned even if the intervention is not based on this theory | |  |
| **2** | **Targeted construct mentioned as predictor of behavior** | | “Targeted” construct refers to a psychological construct that the study intervention is hypothesized to change | |  |
| **3** | **Intervention based on single theory** | | The intervention is based on a single theory (rather than a combination of theories or theory and predictors) | |  |
| **4** | **Theory or predictors used to select recipients for the intervention** | | Participants were screened or selected based on achieving a particular score or level on a theory-relevant construct or predictor | |  |
| **5** | **Theory or predictors used to select or develop intervention techniques** | | The intervention is explicitly based on a theory or predictor or combination of theories and predictors | |  |
| **6** | **Theory or predictors used to tailor intervention techniques to recipients** | | The intervention differs for different subgroups that vary on a psychological construct or predictor at baseline | |  |
| **7** | **All intervention techniques are explicitly linked to at least one theory-relevant construct or predictor** | | Each intervention technique is explicitly linked to at least one theory-relevant construct or predictor | |  |
| **8** | **At least one, but not all, of the intervention techniques are explicitly linked to at least one theory-relevant construct or predictor** | | At least one, but not all, of the intervention techniques are explicitly linked to at least one theory-relevant construct or predictor | |  |
| **9** | **Group of techniques are linked to a group of constructs or predictors** | | A cluster of techniques is linked to a cluster of constructs predictors | |  |
| **10** | **All theory-relevant constructs or predictors are explicitly linked to at least one intervention technique** | | Every theoretical construct within a state theory, or every stated predictor, is linked to at least one intervention technique | |  |
| **11** | **At least one, but not all, of the theory-relevant constructs or predictors are explicitly linked to at least one intervention technique** | | At least one, but not all, of the theoretical constructs within a stated theory or at least one, but not all, of the stated predictors (see I5) are linked to at least one intervention technique | |  |
| **12** | **Measure of theory-relevant constructs** | | | |  |
|  | 12a | Theory-relevant constructs are measured: post intervention | | At least one construct of theory (or predictor) mentioned in relation to the intervention is measured post intervention | |
|  | 12b | Theory-relevant constructs are measured: post and pre intervention | | At least one construct of theory (or predictor) mentioned in relation to the intervention is measured pre and post intervention | |
| **13** | **Changes in measured theory-relevant constructs or predictors** | | The intervention leads to significant change in at least one theory-relevant construct or predictor (vs control group) in favor of the intervention | |  |
| **14** | **Mediational analysis of constructs or predictors** | | | |  |
|  | 14a | Mediator predicts the dependent variable | | Mediator predicts dependent variable, or change in mediator leads to change in dependent variable | |
|  | 14b | Mediator predicts dependent variable, controlling for the independent variable | | Mediator predicts dependent variable when controlling for independent variable | |
|  | 14c | Intervention does not predict the dependent variable when controlling the independent variable | | Intervention does not predict dependent variable when controlling for mediator | |
|  | 14d | Mediated effect is statistically significant | | Mediated effect is statistically significant | |
| **15** | **Results discussed in relation to theory** | | Results are discussed in terms of the theoretical basis of the intervention | |  |
| **16** | **Appropriate support for theory** | | Support for the theory is based on appropriate mediation, or refutation of the theory is based on obtaining appropriate null effects (ie, changing behavior without changing the theory-relevant constructs) | |  |
| **17** | **Use of results to refine theory** | | | |  |
|  | 17a | Results used to refine theory: adding or removing constructs to the theory | | Authors attempt to refine the theory upon which the intervention was based by adding or removing constructs to the theory | |
|  | 17b | Results used to refine theory: specifying that the interrelationships between the theoretical constructs should be changed | | Authors attempt to refine the theory upon which the intervention was based by specifying that the interrelationships between the theoretical constructs should be changed and spelling out which relationships should be changed | |
