# Supplementary material for: Effect of implementation interventions on nurses’ behaviour in clinical practice: a systematic review, meta-analysis and meta-regression protocol
Source: Syst Rev. 2019 Dec 5;8:305. doi: 10.1186/s13643-019-1227-x (PMC6896305; doi:10.1186/s13643-019-1227-x)
Supplement: Supplementary file 6 — Additional file 6. List of Mechanisms of ActionR1. [file 13643_2019_1227_MOESM6_ESM.docx]

**ADDITIONAL FILE 6**

**List of Mechanisms of Action**

- Source: Carey, R. N., Connell, L. E., Johnston, M., Rothman, A. J., de Bruin, M., Kelly, M. P., & Michie, S. (2018). Behavior change techniques and their mechanisms of action: a synthesis of links described in published intervention literature. *Annals of Behavioral Medicine*. doi: 10.1093/abm/kay078

| **#** | **Mechanism of Action** | **Definition** |
| --- | --- | --- |
| **1** | **Knowledge** | An awareness of the existence of something. |
| **2** | **Skill** | An ability or proficiency acquired through practice. |
| **3** | **Social/Professional Role & Identity** | A coherent set of behaviours and displayed personal qualities of an individual in a social or work setting. |
| **4** | **Beliefs about capabilities** | Beliefs about one’s ability to successfully carry out a behaviour. |
| **5** | **Optimism** | Confidence that things will happen for the best or that desired goals will be attained. |
| **6** | **Beliefs about consequences** | Beliefs about the consequences of a behaviour (i.e. perceptions about what will be achieved and/or lost by undertaking the behaviour, as well as the probability that a behaviour will lead to a specific outcome). |
| **7** | **Reinforcement** | Processes by which the frequency or probability of a response is increased through a dependent relationship or contingency with stimulus or circumstance. |
| **8** | **Intention** | A conscious decision to perform a behaviour or a resolve to act in a certain way. |
| **9** | **Goals** | Mental representations of outcomes or end states that an individual wants to achieve. |
| **10** | **Memory, attention & decision processes** | Ability to retain information, focus on aspects of the environment and choose between two or more alternatives. |
| **11** | **Environmental context & resources** | Aspects of a person’s situation or environment that discourage or encourage the behaviour. |
| **12** | **Social influences** | Those interpersonal processes that can cause oneself to change one’s thoughts, feelings or behaviours. |
| **13** | **Emotion** | A complex reaction pattern involving experiential, behavioural, and psysiological elements. |
| **14** | **Behavioural regulation** | Behavioural, cognitive and/or emotional skills for managing or changing behaviour. |
| **15** | **Norms** | The attitudes held and behaviours exhibited by other people within a social group. |
| **16** | **Subjective norms** | One’s perceptions of what most other people within a social group believe and do. |
| **17** | **Attitude towards the behaviour** | The general evaluations of the behaviour on a scale ranging from negative to positive. |
| **18** | **Motivation** | Processes relating to the impetus that gives purpose or direction to behaviour and operates at a conscious or unconscious level. |
| **19** | **Self-image** | One’s conception and evaluation of oneself, including psychological and physical characteristics, qualities and skills. |
| **20** | **Needs** | Deficit or something required for survival, well-being or personnel fulfilment. |
| **21** | **Values** | Moral, social or aesthetic principles accepted by an individual or society as a guide to what is good, desirable or important. |
| **22** | **Feedback processes** | Processes through which current behaviour is compared against a particular standard. |
| **23** | **Social learning/imitation** | A process by which thoughts, feelings and motivational states observed in others are internalized and replicated without the need for conscious awareness. |
| **24** | **Behavioural cueing** | Processes by which behaviour is triggered from either the external environment, the performance of another behaviour, or from ideas appearing in consciousness. |
| **25** | **General attitudes/beliefs** | Evaluations of an object, person, issue or concept on a scale ranging from positive to negative. |
| **26** | **Perceived susceptibility/vulnerability** | Perceptions of the likelihood that oneself is vulnerable to change. |
